# The Relationship between Nuchal Cord and Adverse Obstetric and Neonatal Outcomes: Retrospective Cohort Study

**DOI:** 10.3390/pediatric14010007

**Published:** 2022-01-24

**Authors:** Marta Młodawska, Jakub Młodawski, Grzegorz Świercz, Rafał Zieliński

**Affiliations:** Collegium Medicum, Jan Kochanowski University in Kielce, 25-369 Kielce, Poland; jakub.mlodawski@ujk.edu.pl (J.M.); swierczag@poczta.onet.pl (G.Ś.); rafal.zielinski@ujk.edu.pl (R.Z.)

**Keywords:** nuchal cord, neonatal outcome, umbilical cord, obstetrics outcome, gas analysis

## Abstract

Objective: The twisting of the umbilical cord around the fetal neck is a common phenomenon in the delivery room, and despite the lack of univocal evidence of its negative impact on perinatal events, it causes anxiety and stress in patients. The aim of the study was to assess the prevalence of nuchal cord and its impact on adverse obstetric and neonatal outcomes. Methods: We conducted a retrospective cohort study. All patients who gave birth in the clinic within one year (n = 1467) were included in the study group. We compared the prevalence of nuchal cord in distinct subgroups of patients. In the next stage, we estimated the chance of specific perinatal outcomes and compared the neonatal outcomes between groups with and without nuchal cord. Results: Nuchal cord was present in 24% of labors. It was twice as common among patients giving birth vaginally (32.14%) than among patients giving birth by a caesarean section (16.78%, *p* < 0.001). Nuchal cord was also more frequent in births with meconium-stained amniotic fluid (33.88% vs. 23.34%, *p* = 0.009). In the group of patients with nuchal cord, we observed a slight increase in the risk of a non-reassuring fetal heart rate trace (OR = 1.55, CI 95% 1.02–2.36) as an indication of the completion of labor by caesarean delivery. We did not note an increase in the risk of completing natural childbirth by vacuum extraction. In the group of nuchal cord patients, there was a higher chance of a serious or moderate neonatal condition in the first minute of life (Apgar 0–7 points) (OR = 2.00, 95% CI = 1.14–3.49). Conclusions: Nuchal cord increases the risk of a caesarean delivery due to a non-reassuring fetal heart rate trace. Nuchal cord increases the chance of a reduced Apgar score (0–7 points) in the first minute of life. The observed relationships do not translate to neonatal arterial blood gas testing.

## 1. Introduction

Nuchal cord (NC), i.e., the twisting of the umbilical cord around the fetal neck, is a common phenomenon in the delivery room. In obstetrics, there are also cases of the umbilical cord wrapping around another part of the body, such as the fetal torso or limbs; however, due to international nomenclature, in this study, only cases of umbilical cord wrapping around the fetal neck will be treated as NC. The incidence of any NC at birth increases with gestational age and is estimated at 19–24% [1]. A single loop around the neck is a more common phenomenon than multiple loops (16%, 3%, 1%, and <1% for single, double, triple, and quadruple NC loops, respectively) [1]. Most often, NC has no clinical impact on the condition of the newborn after delivery. Single cases of tight nuchal cord (due to the lack of other risk factors) can theoretically be associated with clinical consequences, such as death, birth asphyxia, emergency Caesarean birth, or neurological complications. However, it is often impossible to prove that NC is the cause of the above-mentioned outcomes. Numerous myths concerning the occurrence of NC have arisen both among patients and medical staff. Late pregnancy complications are often explained by NC. Moreover, patients frequently ask sonographers about the presence of the umbilical cord around the fetal neck. Despite unknown clinical implications, the awareness of its occurrence may cause anxiety and stress among patients [2]. Ultrasound screening for NC and including such information in the description of ultrasounds during pregnancy and labor is currently not recommended [3,4].

## 2. Objective

The aim of the study was to evaluate the prevalence of NC, as well as its impact on different perinatal outcomes, including the clinical condition of the neonate after delivery, umbilical cord blood gas parameter values, and delivery method. We hope that the analysis of medical records of almost 1.5 patient cohorts will complete the current knowledge about NC.

## 3. Material and Methods

We conducted a retrospective cohort study. We analyzed the medical records of all patients who gave birth at the Department of Obstetrics and Gynecology of the Provincial Combined Hospital in Kielce in 2018. Patients who gave birth at <37 Hbd and those with multiple pregnancies, as well as patients with no information on NC (its presence or absence), were excluded from the study. The study was approved by the bioethics commission at the Jan Kochanowski University in Kielce according to resolution No. 2/2021 of 12 January 2021.

A total of 1467 patients were included in the study group. The demographic characteristics of the group are presented in Table 1. NC was defined as the presence of an umbilical cord wrapped at least once around the neck of the fetus at the time of delivery of the fetal head in the case of vaginal delivery (VD) or at the time of fetal extraction in the case of caesarean delivery (CD). We compared the prevalence of NC in distinct subgroups of female patients. In the next stage, by extrapolating the prevalence of NC a priori (i.e., before the onset of labor), we estimated the risk of specified perinatal events and compared the neonatal outcomes between groups with and without NC. The umbilical cord blood gas analysis was conducted using the ABL800FLEX apparatus. Statistical analysis was performed by employing Statistica 13.1 (Tibco Software) and RStudio (ver. 1.2.1335) software. In the case of continuous variables, we presented medians as a measure of central tendency, whereas the interquartile range was employed as a measure of dispersion due to the inability to fulfil the presumption concerning a close to normal distribution. The continuous variables among groups were compared using the Mann–Whitney U test. In the case of qualitative variables, we presented the data as a percentage of events in a specific group. The groups were subsequently compared by employing the Pearson’s χ2 test. Yates’s correction was applied for small expected values. In order to estimate the relative risk, we calculated the odds ratios (OR) and 95% confidence intervals (CI 95%) using univariate analysis. We assumed that the significance level was α = 0.05.

## 4. Results

The prevalence of NC in respective patient subgroups is summarized in Table 2. It is noteworthy that NC was nearly twice as common among patients having natural birth (32.14%) as among patients who gave birth by caesarean section (16.78%) (*p* = 0.000). NC also occurred more commonly in patients with meconium-stained amniotic fluid (MSAF) (33.88% vs. 23.34%, *p* = 0.009). The parity, mode of CD (planned, intrapartum), vacuum extraction (VE) delivery compared to vaginal (non-VE) delivery, time of delivery (delivery before and after due date), and sex of the neonate made no difference in the prevalence of NC. The mean age of the patients with NC did not differ from the non-NC group (31.4 vs. 32.1 years, *p* = 0,61). The risk of the occurrence of obstetric events and the comparison of neonatal outcomes in the presence and absence of NC are presented in Table 3. It is noteworthy that, in the group of patients with NC, there was a slight increase in the risk of a non-reassuring fetal heart rate (NRFHR) (OR = 1.55, 95% CI 1.02–2.36) as an indication of delivery by CD. An increase in the risk of ending vaginal delivery by VE was not noted. The median pH values and the percentage of neonates born with an umbilical cord blood pH of <7.2 and 7.1 did not vary between the groups. In the group of patients with NC, there was a significant risk of the clinical condition of the neonate, in the first minute of life, to be assessed as serious or moderate on the Apgar scale (0–7 points) (OR = 2.00, 95% CI = 1.14–3.49). There was no significant difference with the presence of NC in the Apgar score at the 5th minute of life.

## 5. Discussion

The wrapping of the umbilical cord around the entire circumference (360 degrees) of the fetal neck can be described as tight or loose, single or multiple, and can occur in two ways. Specifically, the placental end of the umbilical cord can run over the umbilical end during wrapping (a safer option that can result in unwrapping with fetal movements), or it can run under the umbilical end (no possibility of spontaneous unwrapping, the risk of a true umbilical cord knot is high). The wrapping of the umbilical cord around the fetal neck is an accidental event; however, the risk of NC is increased by excessive fetal movements or by an excessive length of the umbilical cord [5,6]. The risk of umbilical cord wrapping around the fetal neck increases with the duration of pregnancy, whereas there is no relation between the frequency of this complication and the age of the mother or her race [7]. The frequency of entanglement has been reported to increase linearly throughout gestation [8]. During our study, NC was diagnosed in 24% of patients, which is consistent with the findings of the latest meta-analysis, which included over 270,000 births [1]. Ultrasound with color Doppler imaging enabled us to correctly identify 72% of single and 94% of multiple NC cases, with the greatest sensitivity after 36 weeks (93% vs. 67% before 36 weeks of gestation) [9]. The occurrence of NC between consecutive ultrasound scans varies due to fetal movement. Only in 37% of patients diagnosed with NC in the second or third trimester of pregnancy is the diagnosis confirmed during the perinatal period. In 15% of patients without a previous diagnosis, NC will be diagnosed only at the time of delivery [10]. There is a possibility of NC resolving spontaneously; however, this is less likely in fetuses being delivered on their due date or when the umbilical cord is wrapped around the fetal neck several times [5]. Due to the lack of sufficient evidence for increased adverse fetal outcomes, as well as the occurrence of significant anxiety in the mothers and the carrying out of unnecessary tests and medical appointments [2], the NC assessment is not included in the ultrasound screening [3,4]. In a situation where the patient asks a direct question about the umbilical cord being wrapped around the fetal neck during ultrasound examination, she should be reassured and informed that the visibility of NC is treated as a variant of the norm and does not affect any proceedings during pregnancy [3,4,11]. Nevertheless, in the case of the umbilical cord wrapping around the fetal neck three times, some clinicians recommend increased ultrasound monitoring involving a color Doppler examination of the fetal vascular flow [12].

Despite the ubiquitous opinion about the negative impact of NC on the condition of the fetus or neonate, to date, the data correlating NC with obstetric complications are very limited due to methodological reasons. The cause of death due to NC is not fully understood and is suspected to be multifactorial. One potential mechanism may be death by strangulation, in which the primary mechanism in the fetus involves the constriction of the arteries supplying blood to the brain (airway obstruction, also present in intrauterine strangulation, does not play a significant role). The second potential mechanism may be the constriction of umbilical vessels resulting from NC; in particular, the vulnerable thin-walled umbilical veins. NC as the sole cause of death is rare. This may be indicated by individual case reports in which the child’s death could not be explained by any other cause [13], as well as by symptoms, such as ecchymosis on the facial skin, neck, or in the conjunctiva of the eyes [14,15] suggesting strangulation as the cause of death. A 2020 study on a group of nearly a quarter of a million patients showed no differences in mortality between patients diagnosed with NC vs. those without NC diagnosis [16]. An increased risk of fetal or neonatal death in the event of intrauterine NC was also not indicated by several other large retrospective studies [1,8,13,17], even in tight nuchal cord situations [18]. A 2020 meta-analysis including 145 studies showed an increased risk of stillbirth in the event of true umbilical cord knots (OR 4.65, 95% CI 2.09, 10.37) [1].

Rotations performed by the fetus during vaginal delivery may increase the degree of pressure on the umbilical cord, causing a decrease in the fetal heart rate (FHR). Some studies revealed that only umbilical cord wrapping involving multiple loops around the fetal neck increased the risk of NRFHR [19]. Other studies demonstrated that even a single loop around the fetal neck was associated with an increased risk of NRFHR during labor (adjusted OR 1.61, 95% CI 1.55–1.68) [20,21]; however, as the authors point out, these fluctuations may at least partially be a result of a more frequent use of induction methods during delivery with NC vs. the control group [20,21]. Nonetheless, in the above-mentioned studies, the occurrence of NRFHR did not lead to an increased risk of CD [21]. This is inconsistent with the previously mentioned study from 2020, which involved 243,682 deliveries. In this study, the increase in the abnormal fetal heart rate in the NC group (11.6% vs. 4.9%, *p* < 0.01) probably contributed to a higher rate of emergency CD vs. elective CD in the NC group (15.9% vs. 11.7%, *p* < 0.01) [16]. Additionally, in the above research, the NC group exhibited a higher risk of MSAF (17.3% vs. 14.3%, *p* < 0.01) and assisted delivery (4.0% vs. 3.1%, *p* < 0.01). Analogously, our results indicate a more frequent occurrence of MSAF in the NC group (33.88% vs. 23.34%, *p* = 0.009), an increased risk of CD due to non-reassuring CTG trace in the NC group (OR = 1.55, CI 95% 1.02–2.36), but no increased risk of natural birth involving VE. According to the literature, NC is statistically less common in CD [16,21], which aligns with our results. Specifically, NC was nearly twice as likely in vaginal delivery patients as in CD patients (32.14% vs. 16.78%, respectively, *p* = 0.000). These data suggest that the movements of the fetus during natural labor may contribute to the development of NC, and in the case of CD performed mainly before the onset of labor, there is a lower incidence of NC [16,21].

In our analysis, the incidence of NC increased the risk of a moderate and serious clinical condition of the neonate, which was assessed using the Apgar score within the first minute of life. Similar results of a reduced Apgar score within 1 min of life in the group of children with NC vs. the control group, not observed at 5 min of life, were shown by Sheiner et al. [21], Schäffer et al. [11], and Spellacy et al. [22]. However, the literature data conflict. There are studies showing no increased risk of a reduced Apgar score at both the 1st and 5th minute of life [16,23]. In our study, the percentage of neonates born with an umbilical cord blood pH of <7.2 and 7.1 did not differ among the groups, which is inconsistent with the conclusions drawn by Schäffer et al., who noted that unfavorable neonatal arterial cord blood gas values were observed more frequently in the group of children with NC [11]. This may be a result of the method employed for collecting the umbilical cord blood. Venous blood collected at our clinic, in the case of perinatal asphyxia, may not depict the changes in the fetal circulation as reliably as arterial blood. Martin et al. [24] demonstrated that the Apgar score and gasometric parameters obtained from the umbilical vein did not differ between the group of children with NC and the control group. However, dissimilarities were noticed in terms of the gasometric parameters obtained from the umbilical artery. Specifically, a lower pH, lower oxygen content, and higher PCO2 levels were observed. Gosh et al. did not show an increased risk of an umbilical vein pH of <7.20, umbilical vein base excess of −11, umbilical artery pH of 7.1, or umbilical vein base excess of −11 in the group of children with NC [23].

There is also no strong clinical data supporting the claim that NC is associated with setbacks in psychomotor development. Symptomatic NC, which is identified before labor as being extremely tight or having multiple loops, may be associated with a subclinical deficit in the neurodevelopmental performance at 1 year of age [25]. It is methodologically challenging to associate NC with cerebral palsy (CP) due to the lack of NC screening, significant NC prevalence, and complex nature of CP [26]. A large amount of conflicting evidence concerning this complication can be found in the literature. A large retrospective study, including more than 240,000 singleton deliveries, did not reveal an increased risk of CP in the NC group [27]. Meanwhile, a population-based case–control study demonstrated that the wrapping of the fetal neck with the umbilical cord was associated with a 2.8-fold increase in the risk of spastic cerebral palsy in newborns with nuchal cord (OR 2.8, 95% CI 1.31–6.02) [28]. It is possible that prolonged hypoxia and acidosis might result from tight NC. A retrospective study indicated an increased risk of unexplained spastic quadriplegia (OR 18, 95% CI 6.2–48) in a group of children with tight NC [29], whereas another study showed no significant correlation between tight NC and CP [28]. Tight NC is an independent neonatal hypoxic–ischemic encephalopathy (HIE) factor [30]. NC does not increase the risk of other long-term complications, such as cardiovascular or respiratory morbidities [16].

We recognize the limitations of our study. All problems associated with retrospective data analyses certainly apply to this report. In addition, the number of loops of the umbilical cord around the fetal neck was not analyzed as a variable because we found this to be charted inconsistently. Furthermore, the occurrence of NC was assessed only after delivery. However, due to the fact that the percentage of NC diagnosed after delivery is comparable to that of NC assessed using color Doppler ultrasound at the time of delivery (the sensitivity in prospective studies ranges from 83% to 97%) [31,32], the data could be extrapolated in this manner.

## 6. Conclusions

1. The incidence of NC slightly increases the risk of ending a pregnancy with a CD due to a non-reassuring fetal heart rate trace and does not increase the risk of natural birth by VE;
2. NC is associated with an increased risk of a reduced Apgar score (0–7 points) in the first minute of life;
3. However, the presence of NC does not increase the risk of a decreased pH of the umbilical cord blood.

## Figures and Tables

**Table 1:** Demographic characteristics of the group.

	Study Group (n = 1467)
age (median, IQR)	32 (7)
gestational age at delivery (median, IQR)	39 (2)
pluriparas (%)	51% (n = 748)
epidural analgesia (%)	8.24% (n = 121)
cesarean delivery (cd) rate (%)	52.4% (n = 769)
elective cd (%)	46.68% (n = 410)
intrapartum cd (%)	53.32% (n = 359)
vacuum extraction (%)	1.97% (n = 29)
delivery after completed 40 weeks of gestation (%)	32.71% (n = 480)
nuchal cord present (%)	24% (n = 353)

**Table 2:** Prevalence of nuchal cord in individual subgroups of patients. [CD—caesarean delivery, MSAF—meconium stained amniotic fluid, GA—gestational age].

	Nullipara	Pluripara	*p*
nuchal cord present (%)	25.32%	22.88%	*p* = 0.27
	vaginal delivery	cesarean delivery	
nuchal cord present (%)	32.14%	16.78%	*p* = 0.000
	elective CD	intrapartum CD	
nuchal cord present (%)	15.60%	17.80%	*p* = 0.41
	vaginal delivery (non VE)	vacuum extraction (VE)	
nuchal cord present (%)	31.71%	47.37%	*p* = 0.14
	non-MSAF	MSAF	
nuchal cord present (%)	23.34%	33.88%	*p* = 0.009
	GA of 37–39 weeks	GA beyond 40 weeks	
nuchal cord present (%)	22.70%	26.80%	*p* = 0.07
	female newborn	male newborn	
nuchal cord present (%)	45.45%	54.55%	*p* = 0.28

**Table 3:** The risk of specific obstetric and neonatal events depending on the prevalence of nuchal cord. The comparison of the cord blood pH in both groups. [NRFHR—non-reassuring fetal heart rate, CD—caesarean delivery].

	Nuchal Cord Present			
	No	Yes	*p*	OR (95% CI)
NRFHR as indication of CD (n = 1108 elective CD excluded)	8.64%	12.79%	0.04	OR = 1.55(1.02–2.36)
vacuum extraction (n = 69—CD excluded)	2.11%	4.02%	0.14	OR = 1.93 (0.77–4.83)
pH < 7.1	0.18%	0.57%	0.22	OR = 3.16 (0.44–22.53)
pH < 7.2	1.53%	2.55%	0.2	OR = 1.68 (0.74–3.81)
1st minute Apgar score 0–3	0.36%	0%	0.25	N/A
1st minute Apgar score 0–7	3.07%	5.97%	0.01	2.00 (1.14–3.49)
pH (n = 1467) (median, IQR)	7.373 (0.05)	7.375 (0.07)	0.56	N/A
pH—vaginal delivery group (median, IQR)	7.369(0.085)	7.367(0.102)	0.33	N/A
pH—cesarean delivery group (median, IQR)	7.375 (0.005)	7.374 (0.05)	0.75	N/A
pH—intrapartum CD group (n = 408) (median, IQR)	7.373 (0.05)	7.374 (0.065)	0.71	N/A

## Data Availability

The datasets analysed during the current study are available in the public repository, doi:10.17605/OSF.IO/EQV7P.
